# Prostate cancer, chronic myelogenous leukemia and multiple myeloma in a single patient: a case report and review of the literature

**DOI:** 10.1186/s13256-023-03753-z

**Published:** 2023-02-05

**Authors:** Abolghasem Allahyari, Amirhosein Maharati, Amir Masoud Jafari-Nozad, Alireza zangooie

**Affiliations:** 1 Department of Hematology-Oncology, Faculty of Medicine, Mashhad University of Medical Sciences, Mashhad, Iran; 2 Student Research Committee, Mashhad University of Medical Sciences, Mashhad, Iran; 3 Student Research Committee, Birjand University of Medical Sciences, Birjand, Iran; 4 Cellular and Molecular Research Center, Birjand University of Medical Sciences, Birjand, Iran

**Keywords:** Triple primary cancer, Prostate cancer, Chronic myelogenous leukemia, Multiple myeloma, Case report

## Abstract

**Background:**

Synchronous or metachronous multiple primary malignancies (MPMs) are a known phenomenon. These occurrences may be spontaneous or related to environmental risk factors or genetic predisposition. Chronic myelogenous leukemia (CML) and Multiple myeloma (MM) are two uncommon hematologic malignancies, arises from two different cell lineage. The coexistence of CML and MM that is a rare phenomenon, with only 29 cases reported in the literature. To the best of our, this combination of triple primary cancers has not been reported in a single patient.

**Case presentation:**

Herein, we reported a case of an 85-year-old Iranian male with three confirmed primary malignant neoplasms. The patient presented with synchronous prostate cancer and CML, in august 2016. He received imatinib and nilotinib for CML and hormonal therapy for prostate cancer. He remained in good control at further follow-ups for about 5 years. In the follow-up period and after 61 months treatment with tyrosine kinase inhibitors (TKIs), CML was undetectable in molecular tests, but the presence of serum M-protein, abnormal plasma cells in the bone marrow, and CRAB criteria was compatible with MM.

**Conclusion:**

We must evaluate the possibility of multiple primary cancers during cancer treatment and follow-up and it may be worthwhile to monitor serum electrophoresis and protein levels in TKIs-treated patients.

## Introduction

Multiple primary malignancies (MPMs) are defined as the presence of more than one cancer in the same individual with different histology and site [1]. Billroth first described MPMs in 1889, and Warren and Gates later defined them as the occurrence of at least two distinct primary tumors in the same patient [2, 3]. Multiple primary synchronous or metachronous cancers incidence has been reported to range from 0.73 to 11.7% in the literature [4]. Although the precise mechanism of MPMPs development is not clear, previous cancer therapy, smoking, and genetic abnormalities have all been identified as potential risk factors [5]. Developments in tumor screening and treatment have improved cancer survival rate, which has increased the appearance of multiple primary malignancies [6, 7]. In this paper, we reported the case of an 85-year-old man presenting with prostatic cancer, CML, and multiple myeloma. To our awareness, this constellation of tumors has never been diagnosed in the literature.

## Case report

In August 2016, an 85-year-old Iranian male with hypertension and diabetes mellitus presented with a history of dysuria, hesitancy, and urinary frequency and was found to have a urea level of 52 mg/dl and creatinine level of 1.95 mg/dl. Further investigations with the suspicion of prostate cancer were done on the patient. The Prostate-specific antigen (PSA) level was 8.9 mg/dl and suggested the possibility of prostate cancer. Magnetic resonance imaging (MRI) test showed the normal size of the prostate gland, but peripheral zone lesions were suggestive of prostate cancer. Prostate Imaging Reporting & Data System (PI-RADS) score was 5 (very suspicious). According to the PI-RADS score, direct biopsy from the lesions was recommended for the peripheral zone. The pathologic study of 12 separate samples from different areas of the prostate revealed unilateral adenocarcinoma with a histologic Gleason score of 8 (4 + 4), the malignant neoplastic proliferation of epithelial cells with a monolayer glandular design, invasion of the neighboring tissues, and desmoplastic reaction in five samples. However, the appearance of the normal texture was reported in other samples. In the whole-body scan, a bony lesion in the mid portion of the left femur was described. Hormone replacement therapy was started for him in September 2016.

In June 2017, the patient presented with fatigue, weight loss, and left upper quadrant pain. His Complete blood count test showed a hemoglobin level of 10.7 g/dl, mean corpuscular hemoglobin (MCH) level of 23.9, and mean corpuscular hemoglobin concentration (MCHC) level of 27.9, and an elevated RDW-CV with the value of 19.5. White blood cell (WBC) count of 171.84 10^9^/L and platelet count of 644 10^9^/L revealed Leukocytosis and thrombocytosis. He was suspicion of CML and was asked for quantitative reverse transcription polymerase chain reaction (QRT-PCR). Real-time PCR examination was positive for BCR/ABL P210 fusion gene t (9;22) (q34; q11), which confirmed the CML diagnosis. He started treatment with a TKI, imatinib (400 mg/daily). During the follow-ups, due to severe and symptomatic anemia, generalized edema Erythropoietin (Eprex, Epoetin Alfa^®^) was prescribed for the patient. Due to the treatment failure with imatinib (400 mg − 2 years), the patient was shifted to nilotinib (300 mg − 3 years), a second-generation TKI. After administration of nilotinib, the patient was not symptomatic. Quantitative BCR-ABL1 was negative, and the patient showed a deep molecular response to treatment.

**Fig. 1:**
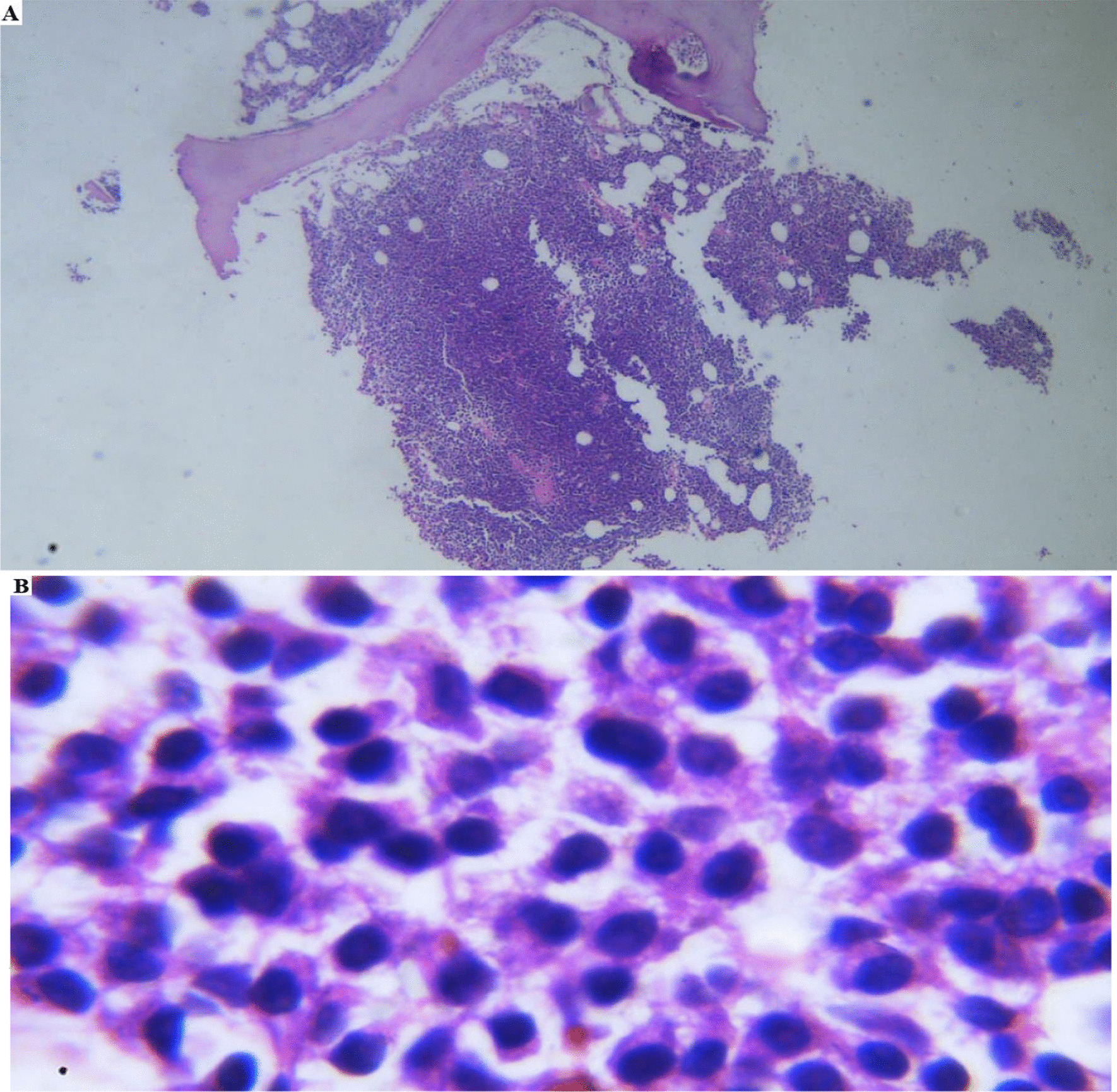
Bone marrow biopsy. **A** Bone marrow biopsy shows hypercellularity, **B** myeloma cells in multiple myeloma in bone marrow biopsy

The patient was followed up for about 5 years. In May 2021, the patient presented with nausea, lethargy, bone pain, weight loss and he had the creatinine level of 4.73 mg/dl, hemoglobin level of 7 g/dl, Erythrocyte sedimentation rate 1st hour level of 104, RBC count of 2.4 × 10^12^/l and Platelet count of 82 × 10^9^/l which was indicative for anemia and renal failure. However, molecular study (quantitative assessment of BCR-ABL1) was still negative and BCR-ABL1 transcript copy was not detected. Further workup revealed a serum total protein level of 8.8 g/dl, albumin level of 3.3 g/dl, immunoglobulin (Ig) G level of 5295 mg/dl, IgA level of 22 mg/dl, IgM level of 10 mg/dl. Serum protein immunotyping (capillary Electrophoresis) detected monoclonal IgG (kappa), which may describe multiple myeloma. Urine protein electrophoresis showed negative detection of Bence Jones kappa to lambda. After injection of 99 m Tc-methylene diphosphonate (MDP), the whole-body bone scan was done with suspicious of prostate cancer bone metastasis. Thoracolumbar scoliosis, degenerative changes in the knee, shoulders and hips was noted. The bony lesion in the mid portion of the left femur were described. Regarding no changes as compared to the previous scans, these findings were not likely due to bone metastasis. Lumbar MRI did not reveal any bony lytic changes. A bone marrow biopsy revealed that 60% of nucleated cells were immature plasma cells which were suggestive of MM (Fig. 1). The Electrodiagnosis (EDX) of the lower limbs showed no SNAPs, reduced CMAPs of bilateral peroneal nerves, and neurogenic pattern on needle EMG. Therefore, the test was abnormal and compatible with chronic sensorimotor distal polyneuropathy with axonal features. We started MP (melphalan and prednisolone) due to his neuropathy. Unfortunately, despite our treatment, he passed away after 6 months.

## Discussion

Multiple primary cancers can be categorized into two groups depending on the time of diagnosis. Metachronous cancers occur sequentially and more than 6 months apart, whereas synchronous cancers happen simultaneously. Synchronous cancers are less prevalent than metachronous cancers. Irimie *et al.* reviewed about one million patients with various cancers and found that the prevalence of Multiple primary cancers was approximately 1.7%. Another study has reported that 0.16% of patients had three or more primary malignant tumors [8]. The most common sites of Multiple primary cancers were breast-gynecologic cancers and head and neck-lung cancers in females and males, respectively [9]. Therefore, the coexistence of two hematologic malignancies from different cell lines with a solid tumor is a rare phenomenon in cancer patient.

Our case was an 85-year-old man diagnosed with triple primary cancers, including CML, prostate cancer, and MM, in which CML and prostate cancer were synchronous. In August 2016, He was an 85-year-old man at the moment of diagnosis with CML and prostate cancer and after 61-month treatment with imatinib he developed another primary cancer, multiple myeloma, based on the presence of serum M-protein, abnormal plasma cells in the bone marrow, and CRAB criteria. Upon reviewing the current literature, the combination of these primary cancers, including prostate cancer, chronic myelogenous leukemia, and Multiple myeloma, has not been reported. Another notable feature of the presented case was that the coexistence of CML and MM that is a rare phenomenon, with only 29 cases reported in the literature (Table 1).

**Table 1:** Cases of synchronous MM and CML recorded in literature so far

Age (Yr)/Sex	Diagnosis interval (month)	MM			CML		Study
		Type	Bone lesion	Chemotherapy	WBC count	Chemotherapy	
CML + MM							
58/M	0	IgGκ	Yes	MP	140	HU, BU TG	[20–22]
72/F	0	IgGκ	Yes	IFN, VDS and PD	162.4	IFN, HU	[20–22]
81/M	0	IgAκ	Yes	MP	28.7	NR	[20–22]
66/M	0	IgGκ	Yes	MP	171	HU, BU, IFN	[20–22]
85/F	0	IgGλ	Yes	NR	8.1	NR	[20–22]
71/F	0	IgGκ	No	MP, BD, Len, Cyc	12.7	IM	[20–22]
64/F	0	IgAκ	No	BD	27.2	IM	[20–22]
88/M	0	IgDκ	No	Len, BD	40	IM	[22]
77/M	0	IgAλ	NR	BR	6.2	NR	[22, 23]
64/M	0	IgAλ	Yes	Thal, Dex	13.1	NT	[24]
MM → CML							
77/M	33	BJP	No	NT	145	6-MP	[20–22]
71/M	24	IgGκ	Yes	MP	40.8	HU	[20–22]
70/M	33	IgGκ	No	NR	25.2	HU	[20–22]
47/M	33	BJPκ	Yes	NR	23.9	NR	[20–22]
68/M	54	IgGλ	Yes	VCR, Dox, Dex, zol	11.81	IM	[21]
62/F	17	IgGκ	No	BD, Cyc, Dox, Len	NR	DA	[20]
60/M	48	IgGκ	Yes	Len, BD	11.2	DA	[22, 25]
51/F	3	IgG	Yes	BD	NR	IM	[26]
76/M	28	IgGκ	Yes	Len, Dex	35.8	DA, BO	[27]
68/M	24	IgAκ	NR	Cyc, Thal	NR	IM	[28]
CML → MM							
65/F	113	IgGκ	Yes	BU, PM	43	BU, PM	[20–22]
71/M	38	BJPλ	NR	NR	NR	IM	[20–22]
68/M	20	IgGλ	No	MP	NR	IFN, IM	[20–22]
76/M	14	IgAλ	No	MP	NR	IFN, IM	[20–22]
57/F	65	IgAλ	NR	Thal, BD, VCR, Dox	52.38	IM	[20–22]
72/F	3	IgGκ	No	NT	31.3	IM	[20–22]
63/F	120	IgGκ	NR	BD, Len	17.8	IM	[21]
76/M	38	IgAλ	No	NT	18.8	IM	[29]
58/M	137	IgGκ	Yes	BD, Len, Thal	NR	IM	[30]
85/M	61	IgGκ	No	MP	171.8	IM, NL	Present case

6-MP: 6-mercaptopurine, BD: bortezomib and dexamethasone, BJP: Bence jones protein, BO: bosutinib, BR: bortezomib, BU: busulphan, CML: chronic myelogenous leukemia, Cyc: cyclophosphamide, DA: dasatinib, Dex: dexamethasone, Dox: doxorubicin, F: female, HU: Hydroxyurea, IFN: interferonalpha, IM: imatinib, Len: lenalidomide, M: male, MM: multiple myeloma, MP: melphalan and prednisolone, NL: nilotinib, NR: not reported, NT: no treatment, PD: prednisolone, PM: phenylalanine mustard TG: thioguanine, Thal: thalidomide, VCR: Vincristine, VDS: Vindesine sulfate

In 10 of 29 previous cases (patients 1–10), CML and MM were diagnosed simultaneously; the diagnosis of MM preceded that of CML in ten cases (patients 11–20), and the diagnosis of CML preceded that of MM in the remaining nine cases (patients 21–29). In the latter nineteen cases, the interval between the diagnosis of each disease ranged from 3 to 137 months. Among previously reported cases, 17 patients had immunoglobulin G myeloma, 8 patients had immunoglobulin A myeloma, 3 patients had light chain disease (Bence Jones protein) and one patient demonstrated immunoglobulin D myeloma. Eight of the nine patients who were first diagnosed with CML had been treated with imatinib mesylate for CML before MM developed. Seven of the ten patients who first developed MM received chemoradiotherapy for MM.

The occurrence of CML and MM in the same patient is unusual; it may suggest a correlation between the two separate hematological malignancies rather than an incidental occurrence. Several different pathophysiologies have been proposed to describe the synchronous or metachronous presence of MM and CML.

Firstly, CML can develop into blastic phase, and in a third of cases, CML blasts transform to lymphoid cells. The transition of CML blasts to lymphoid cells supports the existence of a single stem cell that can differentiate into both myeloid and lymphoid cell lineages. This common stem cell could transform along the lymphoplasmacytic and myeloid lineages, resulting in CML and MM. Whether CML and multiple myeloma share the same genetic risk loci has also been investigated. Although it has been reported that PS0RS1C1-rs2285803 increased the risk of MM in patients diagnosed with CML, but it needs to be further investigated [10]. Even though this hypothesis is appealing, no cases reported in the literature have shown a common cellular origin, and there is no strong evidence to support this hypothesis. Moreover, in our case, when MM was diagnosed, Bcr-Abl mRNA transcript copies were undetectable by PCR, suggesting that CML and MM in our patient had a different cellular origin. Thereby, the etiological correlation of the two diseases cannot be proven by proposing they derive from a common pluripotent stem cell. Nonetheless, the neoplastic transformation could occur at an earlier differentiation stage than the reciprocal translocation between chromosomes 9 and 22.

Secondly, Exposure to cytotoxic drugs and radiation can develop secondary malignancies during the treatment of the first malignancy. In this regard, developing secondary malignancies in the patients treated with melphalan and cyclophosphamide or lenalidomide have been reported. Additionally, it has been shown that patients who received radiation therapy for those prostate cancers showed an increased risk of bladder and colorectal cancer [11]. Nonetheless, our patient did not receive any of mentioned treatments before presenting CML or MM.

In the literature, 8 of the nine patients who first had CML received Imatinib before MM was diagnosed. Moreover, it has been reported that an imatinib-treated patient developed MM after a gastrointestinal stromal tumor [12]. It can be proposed that imatinib mesylate treatment may promote the development of MM. Furthermore, imatinib mesylate stimulates the proliferation of MM cells through activation of the Erk1 and Erk2 mitogen-activated protein kinases (MAPKs). It has also been demonstrated that imatinib blocked MM cell lines proliferation *in vitro* [13]. The relapsed/refractory MM patients revealed no response to imatinib and ended treatment with progressive disease in a clinical trial [14]. Thereby, only nine patients in the literature developed MM among thousands of CML patients receiving imatinib, and this cannot suggest the hypothesis of MM cell acceleration by imatinib. On the other hand, imatinib is a new-generation drug, and its long-term side effects have not been investigated thoroughly. Our patient was the first case who received imatinib and nilotinib for 61 months before the diagnosis of MM, and we cannot prove that the development of MM is a random or accelerated situation. We assume that these TKIs were primarily responsible for this patient’s clinical outcome, but we cannot rule out CML’s potential role, leading to other hematologic malignancies.

Finally, epigenetic alterations of molecular pathways resulting from progression or treatment of primary cancers could potentially pave the ways for other simultaneous malignancies to emerge. For example, DNA damage repair (DDR) alterations, resulting from DDR gene mutations or epigenetic modifications, have been involved in cancer initiation, progression, and treatment response [15]. It has been revealed that DDR genes alterations including BRCA2, BRCA1, CDK12, ATM, FANCD2, or RAD51C affect almost 20% of 333 primary prostate cancer [16]. Another study demonstrated that DDR inhibitors (DDRi s) targeting ATM/ATR/WEE1 checkpoints inducing apoptosis in MM cells [17]. Furthermore, DNA damages are initially sensed by the ataxia telangiectasia mutated (ATM) signal kinase to induce DDR in CML cells [18]. Although we did not examine this hypothesis in our patient, it can be a potential reason for developing multiple primary cancers in a single patient.

In addition, these malignant cells behavior and fate are profoundly influenced by evolving interplay with microenvironment [19], which can affect critical signaling pathways and eventually give rise to other primary malignancies. Other factors such as chronic antigenic stimulation may also be involved.

## Conclusion

Although it is not possible to identify with certainty the cause of the coexistence of MM and CML in our patient, we can conclude that it is a multifactorial condition. However, we could only exclude the hypothesis that the MM originated from a CML hematopoietic stem cell based on the absence of BCR-ABL1 by PCR. TKIs have significantly impacted the CML treatment and is one of the most successful targeted therapies. To the best of our knowledge, there is no correlation between TKIs and MM development, but the long-term side effects should be considered, and it may be worthwhile to monitor serum electrophoresis and protein levels in TKIs-treated patients. Additionally, during cancer treatment and follow-up, we must evaluate the possibility of multiple primary cancers and choose the best treatment according to this possibility.

## Data Availability

The article contains all of the data from this research. If you need any further information, please contact the corresponding author.
